# The role of single fraction Gamma Knife radiosurgery for intraventricular central neurocytomas and the utility of F-18 fluroethyltyrosine: two case reports

**DOI:** 10.1186/s13256-022-03665-4

**Published:** 2022-11-28

**Authors:** Michaela Dedeciusova, John O. Prior, Luis Schiappacasse, David Patin, Marc Levivier, Constantin Tuleasca

**Affiliations:** 1 University of Lausanne (Unil), Faculty of Biology and Medicine (FBM), Lausanne, Switzerland; 2 Department of Clinical Neurosciences, Neurosurgery Service and Gamma Knife Center, Lausanne University Hospital (CHUV), Lausanne, Switzerland; 3 First Faculty of Medicine, Charles University in Prague, Prague, Czech Republic; 4 Department of Neurosurgery and Neurooncology, Military University Hospital Prague, Prague, Czech Republic; 5 Service of Nuclear Medicine and Molecular Imaging, Lausanne University Hospital (CHUV), Lausanne, Switzerland; 6 Radiation Oncology Department, Lausanne University Hospital (CHUV), Lausanne, Switzerland; 7 Institute of Radiation Physics, Lausanne, Switzerland; 8 Signal Processing Laboratory (LTS 5), Ecole Polytechnique Fédérale de Lausanne (EPFL), Lausanne, Switzerland; 9 Centre Hospitalier Universitaire Regional de Lille (Neurooncology and Epilepsy Fellow), Lille, France

**Keywords:** Radiosurgery, Gamma Knife, Central neurocytoma, F-18 FET

## Abstract

**Background:**

Primary treatment of central neurocytomas is surgical resection. Gamma Knife surgery is considered a valuable therapeutic option in case of residual (after subtotal resection) or recurrent central neurocytomas. Here, we focused on the role of F-18 fluroethyltyrosine as a marker to document tumor progression after initial resection, in the context of an atypical central neurocytoma. We also describe MIB-1’s role in evaluating therapeutic decision-making.

**Case presentation:**

Two patients with central neurocytomas were treated by Gamma Knife surgery in our center. The first case (31-year-old Caucasian male) had atypical central neurocytoma. Four and a half years after surgical resection, magnetic resonance imaging and F-18 fluroethyltyrosine documented clear progression of residual central neurocytoma, further treated by Gamma Knife surgery (18 Gy at 50%, target volume 1.4 cc, and prescription isodose volume 1.8 cc). The initial post-Gamma Knife surgery clinical course was uneventful, with progressive volumetric reduction of residual tumor up to 4.5 years, when out-of-field recurrence was suspected and confirmed by local F-18 fluroethyltyrosine hyperactivity. Second single-fraction Gamma Knife surgery was performed (18 Gy at 50%, target volume 0.49 cc, prescription isodose volume 0.72 cc). The second (32-year-old Caucasian female) had previous subtotal resection and typical central neurocytoma. Seven years later, she had residual tumor progression. Single-fraction Gamma Knife surgery was performed (16 Gy at 50% isodose line, target volume 1.7 cc, and prescription isodose volume 2.5 cc). Last follow-up showed tumor volume reduction. Follow-up magnetic resonance imaging showed important volumetric reduction of both treated lesions.

**Conclusions:**

In atypical central neurocytomas, F-18 fluroethyltyrosine could be used as postoperative examination to detect small tumor remnants, follow-up evaluation following the Gamma Knife surgery or, in select cases, following surgical resection. The role of MIB-1 is important in therapeutic decision-making, as tumors with MIB-1 exceeding 2% are characterized by more aggressive clinical course. Single-fraction Gamma Knife surgery remains a valuable therapeutic option for postoperative residual atypical central neurocytomas and central neurocytoma recurrences.

## Introduction

Central neurocytomas (CNs) are rare low-grade neuronal tumors. They encompass only 0.25–0.5% of all brain neoplasms. Central neurocytomas are commonly localized within the lateral or third ventricle, in close proximity to foramen of Monro or attached to septum pellucidum [1, 2]. Current treatment strategies are based on limited available data, including several case reports and retrospective and few prospective case series, as well as meta-analyses [3]. Complete microsurgical resection (CR) is the treatment of choice [4]. Adjuvant radiotherapy (RT) to reduce the recurrence rates in case of subtotal resection (STR) is often advocated [5, 6]. Gamma Knife surgery (GKS) is considered a valuable therapeutic option in case of residual or recurrent CNs [7–10]. The reported 5- and 10-year tumor control rates (TCR) range from 91% to 94%, and from 81% to 91.6%, respectively [8, 11, 12].

Classically, CNs are divided in two variants, which are typical (75%) and atypical (25%). Atypical central neurocytomas (aCNs) present with rapid tumor progression, recurrence, extraventricular extension or even craniospinal dissemination [13, 14]. Atypical CNs are characterized by the MIB-1 labeling index (MIB-1, cell proliferation marker) > 2% or by the presence of focal necrosis, vascular proliferation, or increased mitotic activity [4, 15, 16]. Some evidence exists that MIB-1 greater than 2–3% or atypical histological correlates with worse prognoses and should be further managed by more aggressive primary treatment, including RT or chemotherapy [6, 15, 17, 18].

In the present study, we report two cases of CNs with gross total resection being previously performed. Even though both patients underwent initial microsurgery followed by GKS for tumor recurrence/progression, clinical course of both patients’ variants differed significantly. Two new aspects are addressed: (1) the potential role of F-18 fluroethyltyrosine (F-18 FET) in atypical CNs so as to evaluate tumor progression after initial therapy; and (2) the MIB-1’s value in the context of less versus more aggressive tumors.

## Case report

### Patient selection and design

This is a retrospective historical cohort study. During the period from June 2010 to June 2019, two patients with CNs were treated by GKS in our center. Both patients first underwent surgical resection performed by senior neurosurgeons. According to Swiss regulations, Ethical Committee approval was not necessary, as it was a retrospective review of less than three cases.

### Radiosurgical technique

We applied the Leksell Model G stereotactic frame (Elekta Instruments AB, Sweden) under local anesthesia for both cases. Both patients underwent stereotactic imaging the day of GK. In our center, we use multimodal imaging for target definition, which both patients presented here particularly benefited from: magnetic resonance imaging (MRI, including T1 MPR, T2 SPACE, T2 TSE, T1 MPR with contrast enhancement) and computer tomography (CT). In the case of aCN, F-18 FET was performed shortly before GKS treatment. Both patients were treated with Leksell Gamma Knife Perfexion and ICON (Elekta Instruments, AB, Sweden) by the same operators (ML, CT) during the specified timeframe. Dosimetry planning was performed using Leksell Gamma Plan (LGP version 10.0 and 11.0, Elekta Instruments AB, Sweden).

Follow-up MRI and clinical outpatient visits after GKS were performed up to 6.5 years for case 1 and up to 5 years for case 2. F-18 FET was performed if recurrence of atypical CN was suspected. This was the case for the out-of-field recurrence further described (illustrative case 1), which further benefited from a second GKS (see below).

### Illustrative case 1: atypical CN

**Fig. 1:**
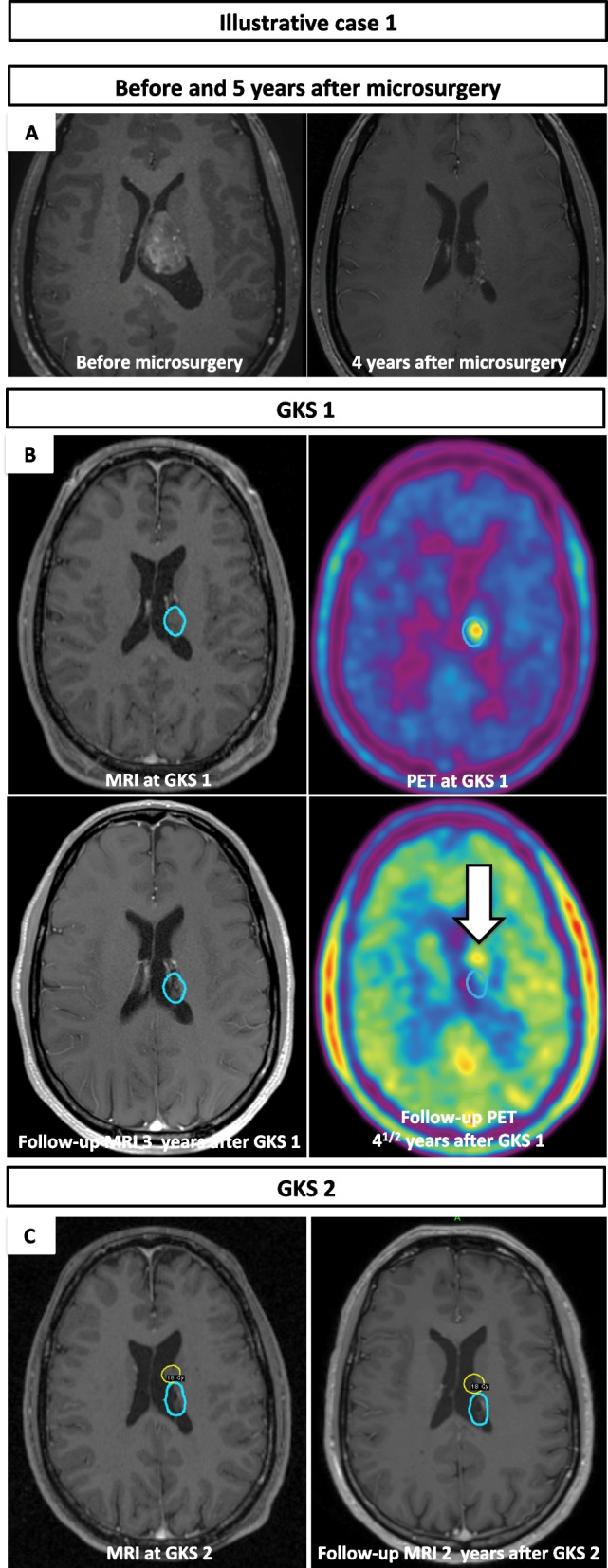
**A** Preoperative and 4 years after microsurgery MRI; **B** MRI and F-18 FET at the time of first GKS (upper image), and 3 years later (lower image); **C** MRI at the time of second GKS (left image) and 2 years later (right), with major shrinkage. Arrow points toward the recurrence which will be further treated by a second GKS

A 31-year-old Caucasian male patient was incidentally diagnosed with left ventricular CN. Two years from initial diagnosis, the patient experienced nausea and vertigo. MRI confirmed volumetric progression and the initial STR was performed via interhemispheric transcallosal approach. The preoperative and postoperative MRI are showed in Fig. 1A. The anatomopathological examination did not show any presence of necrosis or pathological microvascular proliferation. The mean MIB-1 was 5–7%, but focally reaching 10%. Clinical evaluation was unremarkable.

Postoperative MRI revealed possible tumor remnants. There was a discrete capitation on the F-18 FET. Assessment by F-18 FET performed 2 years following the surgery confirmed stability of the residual tumor. Similarly, follow-up MRI documented stability of small tumor remnants for up to 3 years. Both MRI and F-18 FET 4.5 years after surgical resection documented clear progression of residual CN (Fig. 1B). Clinical evaluation was unremarkable.

The tumor recurrence was treated by the GKS (Fig. 1B). The target volume (TV) was 1.4 cc and prescription isodose volume (PIV) was 1.8 cc. Marginal dose prescribed was 18 Gy at the 50% prescription isodose line. The conformity, selectivity, Paddick, and gradient indices were 1.000, 0.778, 0.778, and 2.556, respectively.

The initial post-GKS clinical course was uneventful, with progressive volumetric reduction of residual tumor up to 4.5 years. However, at that time, MRI revealed suspect tumor out-of-field recurrence. The former additionally showed a local capitation on F-18 FET. The second single-fraction GKS was performed (Fig. 1C). The marginal dose was 18 Gy at the 50% prescription isodose. The TV was 0.49 cc and PIV was 0.72 cc. The conformity, selectivity, Paddick, and gradient indices were 1.454, 0.688, 0.688, and 2.636, respectively. The last follow-up control was performed 6.5 years after the first and 2 years after the second GKS, respectively. The patient experienced no adverse radiation effects. Follow-up MRI showed important volumetric reduction of both treated lesions. Clinical evaluation was unremarkable.

### Illustrative case 2: typical CN

**Fig. 2:**
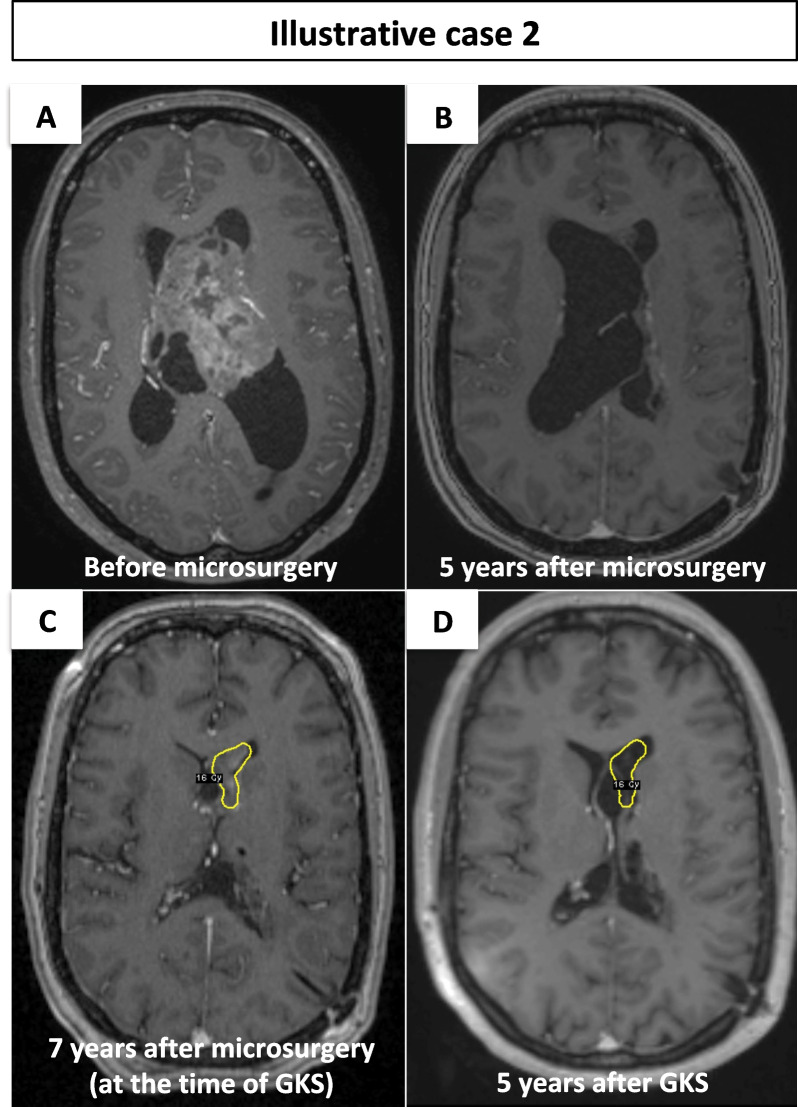
**A** Preoperative MRI; **B** 5 years postoperative MRI; **C** MRI at the time of GKS, showing the dosimetry, which is colored in yellow; **D** MRI 5 years after GKS, with superimposed dosimetry

A 32-year-old Caucasian female patient presented with headaches and gait deviation. MRI revealed voluminous CN of left lateral extending to third ventricle causing obstructive hydrocephalus. The STR via interhemispheric transcallosal approach with the insertion of an external ventricular drain was performed. The preoperative and postoperative MRI is depicted in Fig. 2A. The MIB-1 was in all specimens lower than 3%. Postoperative clinical course was complicated by serious memory deficits, which were later partly ameliorated, with the help of reeducation.

Seven years later, control MRI showed residual tumor progression in anterior part of left lateral ventricle with ventricular enlargement (Fig. 2B). The indication of second surgical resection was not retained because of high risk of memory deficits worsening. Hydrocephalus was managed by the insertion of ventriculoperitoneal (VP) shunt. Tumor progression was treated by the single fraction GKS (Fig. 2C). The maximum marginal dose was 16 Gy at the 50% prescription isodose. The TV was 1.7 cc and the PIV was 2.5 cc. The conformity, selectivity, Paddick, and gradient indices were 1.000, 0.680, 0.680, and 2.800, respectively. The post-GKS clinical course was uneventful. The last follow-up MRI, 5 years after radiosurgery (Fig. 2D), showed reduction of the tumor volume treated by the GKS and global stability of non-treated residual tumor in the third ventricle. There was clinical stability.

## Discussion

In the present manuscript as well as on the basis of previous experience in our institution [19], we used, for the first time, F-18 positron emission tomography (PET) for evaluating small tumor remnants after microsurgical resection or recurrence after radiosurgery (case 1). Several studies have shown a benefit of using FET-PET, as it appears to be superior to fluorodeoxyglucose (FDG)-PET for evaluating and biopsying non-contrast-enhancing brain tumors, specifically WHO grades II and III neoplasms [20].

### Potential role of FDG-PET in the management of atypical CNs

Typically, FDG-PET studies in CNs show lower metabolic rate of glucose compared with the gray matter [21]. To our knowledge the only case of CN with atypical histological features, high MIB-1, and unusually intense FDG uptake was reported by Ohtani *et al*. [22]. The STR with adjuvant conventional RT administering total dose of 50 Gy was performed. Interestingly, shortly after completion of RT, an intense FDG uptake in residual tumor disappeared and FDG-PET was proposed as a potential follow-up examination in atypical CNs [22]. Similarly, in illustrative case 2 of aCN, the suspect discrete hyperactivity on FDG-PET was observed after the surgery, and intense glucose uptake confirmed local recurrence and out-of-field recurrence 4.5 and 9 years later. The comparison of two FDG-PETs (Fig. 2) acquired for the planning of first and second GKS showed a significant metabolic activity decrease within the PIV of first GK.

Importantly, only a few studies containing small patient populations report direct comparisons between FET and FDG-PET for the qualitative and quantitative characterization of brain lesions in humans [19]. A recent meta-analysis suggested a strong advantage of FET-PET over FDG-PET for diagnosis of brain tumors and gliomas [19].

### Other imaging methods showing the high tumor proliferative activity

Not only FDG-PET, but also cerebral metabolic rate of glucose (rCMRGI) and minimum apparent diffusion coefficient (ADCmin), were proposed as markers of high proliferative activity. Mineura *et al*. reported that values of rCMRGl (2.68–6.26 mg per 100 ml per minute) were significantly lower compared with the contralateral gray matter (*P* < 0.02). In contrast to benign course of CNs exhibiting the cold foci, the only one with rCMRGI equivalent to the gray matter presented regrowth 4 months after the STR [21]. Sakamoto *et al*. estimated that the aCNs (MIB-1 LI > 2%) could be differentiated with 100% sensitivity (95% CI 47.8–100%) and 100% specificity (39–100%) if the threshold value of ADCmin is set at 0.55 × 10^–3^ mm^2^ per second (*P* < 0.0001) [23].

### Importance of MIB-1 LI value

Commonly reported MIB-1 cutoff value to be able to differentiate between typical and atypical CNs is 2% [4, 15, 16]. The difference in aggressive CNs, depending on MIB-1 grade, could be also seen by our illustrative cases. In case 1, MIB-1 did not exceed 3%, even focally. Even if STR was performed, progression appeared extremely late; for example, after 7 years. On the contrary, in case 2, MIB-1 LI was 5–7%. Even if this less voluminous tumor was resected almost completely (> 99%), recurrence and out-of-field recurrence were revealed much earlier, at 4.5 and 9 years later, respectively.

The association of MIB-1 LI > 2% with shorter recurrence-free interval was well documented [15, 21, 24]. Rades *et al*. suggested the 3% MIB-1 to be a breakpoint, as reported local failure was 12% in MIB-1 LI ≤ 3% group, as compared to 48% in MIB-1 > 3% group [18]. Similarly, Kaur *et al*. reported the 4-year RR 0% in MIB-1 < 4% group compared with 100% in MIB-1 > 4% group. Moreover, the management by the STR alone leads to 0% recurrence rates in patients with MIB-1 < 4% compared with 100% recurrence rates in patients with MIB-1 > 4% [25].

Following STR, MIB-1 value should guide the next steps within the therapeutic approach. If MIB-1 is low, rather conservative management with close observation should be advocated, avoiding the potential risk (although extremely rare) of adverse radiation events (AREs) after GKS or RT. If the MIB-1 is high, adjuvant GKS should be considered owing to its steep gradient and the possibility of attaining functional preservation [26]. The importance of adjuvant RT following STR of atypical CNs has already been documented in 1997 by Schild *et al*. and later in 2007 by Leenstra *et al*. [6, 27]. The major advantage of adjuvant GKS versus RT is more conformal and selective dose distribution for the first, while performing only one treatment session [28], with possibly less AREs. Retrospectively, the upfront and immediately postoperative adjuvant GKS in case 2 might potentially have avoided the second, out-of-field CN recurrence.

### Gamma Knife surgery in typical versus atypical CNs

GKS is considered a valuable therapeutic option [29] in case of residual or recurrent CNs [7–10]. Satisfactory results of GKS in CNs were documented by several recent series (for example, Yamanaka *et al*. [8], Genc *et al*. [11], Pan *et al*. [30], Karlsson*et al*. [12]). The study cohort included 22–42 patients and median follow-up period ranged from 24 months to 75 months. Median marginal doses ranging from 12 Gy to 16 Gy were prescribed to median tumor volumes from 4.9 ml to 12.6 ml [8, 11, 12, 30]. The 5- and 10-year control rates ranged from 91% to 94%, and from 81% to 91.6%, respectively [8, 11, 12]. Two permanent complications, one intratumoral hemorrhage and one radiation effect, were described by Yamanaka *et al*. [8]. Moreover, Karlsson *et al*. suggest a close imaging monitoring, because 45% of patients developed a partial enlargement of ventricular system [12].

## Conclusion

In our experience, in aCNs, the F-18 FET could be used as postoperative examination to detect small tumor remnants, follow-up evaluation following the GKS/RT, or in select cases following surgical resection. The role of MIB-1 is important in therapeutic decision-making, as tumors with MIB-1 exceeding 2% are characterized by more aggressive clinical course. Single-fraction GKS is a valuable therapeutic option for postoperative residual aCN and CN recurrences, or as upfront treatment in small asymptomatic tumors.

## Data Availability

Data are not available, as besides the radiological images, there is no need for further data.
